# Improved Performance of the QuantiFERON-SARS-CoV-2 Assay with the Extended Set

**DOI:** 10.3390/v15051179

**Published:** 2023-05-17

**Authors:** Lydia Lamara Mahammed, Kahina Bensaid, Sarah Ait-Seddik, Amel Larinouna, Ghania Brahimi, Rosa Belkaid, Ouassila Hamzaoui, Soumia Meriem Rouaki, Cherifa Idder, Ines Allam, Reda Djidjik

**Affiliations:** 1 Immunology Department, Beni-Messous Teaching Hospital, Faculty of Pharmacy, University of Algiers, Algiers 16000, Algeria; 2 Epidemiology Department, Beni-Messous Teaching Hospital, Faculty of Medicine, University of Algiers, Algiers 16000, Algeria; 3 EPSP Cheraga Bouchaoui, Algiers 16000, Algeria; 4 Occupational Medicine, Beni-Messous Teaching Hospital, Faculty of Medicine, University of Algiers, Algiers 16000, Algeria

**Keywords:** QuantiFERON-SARS-CoV-2, COVID-19, cellular immunity, vaccine

## Abstract

Multiple assays have been developed for the characterization of the functional activation of SARS-CoV-2 specific T-cells. This study was conducted to assess the post-vaccination and post-infection T cell response, as detected by the QuantiFERON-SARS-CoV-2 assay using the combination of three SARS-CoV-2 specific antigens (Ag1, Ag2 and Ag3). An amount of 75 participants with different infection and vaccination backgrounds were recruited for the evaluation of humoral and cellular immune responses. An elevated IFN-γ response in at least one Ag tube was observed in 69.2% of convalescent subjects and 63.9% of vaccinated ones. Interestingly, in a healthy unvaccinated case and three convalescents with negative IgG-RBD, we detected a positive QuantiFERON test after stimulation with Ag3. The majority of the T cell responders reacted simultaneously to the three SARS-CoV-2 specific antigens, and Ag3 demonstrated the highest rate of reactivity. At univariable analysis, the only factor that was associated with an absence of a cellular response was time from blood collection, being less than 30 days (OR:3.5, CI95% [1.15–10.50], *p* = 0.028). Overall, the inclusion of Ag3 improved the performance of the QuantiFERON-SARS-CoV-2 and showed a particular interest among subjects who fail to achieve a measurable antibody response after infection or vaccination.

## 1. Introduction

The evaluation of both humoral and cell-mediated response is crucial to monitor the immune protection provided by the natural SARS-CoV-2 infection and the ongoing SARS-CoV-2 vaccination [1,2,3].

As antibody detection is the easiest method to screen, on a large scale, the population for previous infection or vaccination, a lot of investigations have been conducted these last three years toward dissecting the humoral response to SARS-CoV-2. As a result, many reliable standard commercial kits are currently available to detect anti-SARS-CoV-2 antibodies [4,5]. However, several studies have reported decreasing circulating antibodies over time [6,7], and they may be undetectable in patients with asymptomatic or clinically mild disease [8,9]. Evidence also indicated that SARS-CoV-2 variants were able to partially escape humoral response, while cell-mediated immunity is less affected [10,11]. Furthermore, T cell response is more enduring and detectable, even in the absence of seroconversion [8,12,13,14].

Unlike humoral response, the conventional methods to investigate the role of T cells are more complex and require highly specialized facilities. A wide range of either in-house or commercialized tests have been developed for the characterization of the functional activation of SARS-CoV-2 specific T-cells [15]. QuantiFERON-SARS-CoV-2 assay is one of the commercially available IGRA tests (IFN-Gamma Release Assays). The use of this test has been supported by extensive experience acquired in other clinical settings using the same platform, e.g., to evaluate cellular immunity for cytomegalovirus [16] or mycobacterium tuberculosis [17]. Moreover, it is an easy-to-use tool, with the possibility of a routine application. 

The clinical performance of the initial starter set, measuring IFN-gamma (IFN-ɣ) release induced by two spike-derived peptide pools (Ag1 and Ag2) [18], has been evaluated by many authors in different group populations, including immunocompetent and immunocompromised convalescent patients, as well as vaccinated subjects [19,20,21,22,23,24,25,26]. Moreover, a good correlation between cellular responses detected by QuantiFERON-SARS-CoV-2 and ELISpot assays has been demonstrated [27]. Nevertheless, when comparing the QuantiFERON accuracy to a home-made assay, the published data suggested a lower sensitivity for QuantiFERON, likely because of the different peptide composition [23,24]. Thus, an additional antigen (Ag3), covering the S, N, and M domains, and other peptides from proteins encoded by the full genome of SARS-CoV-2, have been included in an extended set to elicit a more complete evaluation of the T-cell-mediated response [28]. However, only a few studies have evaluated the added value of combining the three SARS-CoV-2 specific antigens to assess the T-cell-mediated response [27,29,30,31]. 

From this perspective, the present study was conducted to characterize the post-vaccination and post-infection T-cell responses, as detected by the QuantiFERON-SARS-CoV-2 assay using the combination of the three SARS-CoV-2 specific antigens.

## 2. Materials and Methods

### 2.1. Study Population

**Figure 1:**
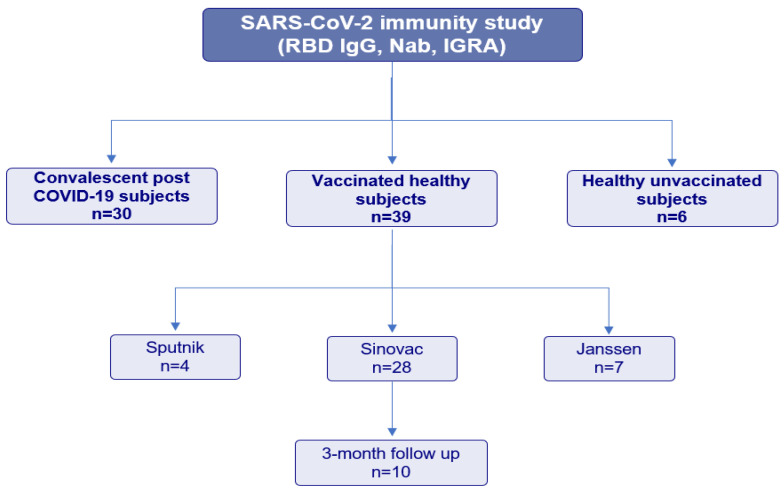
Flow chart of patients’ enrollment. RBD: anti-receptor binding domain. Nab: neutralizing antibodies. IGRA: IFN-Gamma Release Assay.

In this prospective study, we included three groups of subjects (Figure 1):

- Convalescent subjects (*n* = 30, mean age 32 ± 10.7, range (19–57) years, 14 males and 16 females): individuals with previous COVID-19 infection with a positive nasopharyngeal swab and clinically symptomatic (mild to moderate symptoms). None of the participants had been vaccinated before blood drawing. The median time between PCR-confirmed COVID-19 diagnosis and plasma samples was 28 days (range between 17 and 59 days).
- Vaccinated healthy subjects (*n* = 39, mean age 42.9 ± 15, range (18–64) years, 17 males and 22 females): SARS-CoV-2 naïve individuals with no history of COVID-19 symptoms or positive SARS-CoV-2 test who had been fully vaccinated with one of the COVID-19 vaccines available in our country at the time of the study: Sinovac (*n* = 28), Janssen (*n* = 7), or Sputnik (*n* = 4). Subjects were considered fully vaccinated if they received the second dose of the homologous two-dose vaccine regimen (for Sinovac and Sputnik) or a dose of the one dose vaccine (Janssen) without receiving a booster dose. At the first blood collection, the median time from completed vaccination was 32 days (range (16–55) days). Ten patients out of this group (vaccinated with Sinovac) agreed for a second follow-up three months after the second dose of the vaccine (median 97, range (88–108) days).
- Healthy, unvaccinated subjects (*n* = 6, mean age 43.6 ± 14.2, range (19–57) years, three males and three females): SARS-CoV-2 naïve and unvaccinated individuals were included as a negative control group.

All subjects were ≥18 years of age, and enrollment exclusion criteria for all the groups were: HIV infection, immunosuppressive treatment, and immune-related disease that may affect the immune response.

Clinical and demographic information were collected at enrollment in the Department of Medical Immunology of Beni-Messous Teaching Hospital Center in Algiers, Algeria, between November 2021 and April 2022.

All participants signed a written informed consent, and the study was approved by the institutional ethics committee of Beni-Messous Teaching Hospital center, Algiers, Algeria.

### 2.2. Methods

Blood samples were taken for the evaluation of humoral and cellular immune responses. 

#### 2.2.1. Humoral Immune Response

Humoral response to SARS-CoV-2 was evaluated using two essays, which were performed according to the manufacturer’s instructions. 

The quantitative determination of anti-receptor binding domain (RBD) IgG was performed using a chimiluminescent essay (MAGLUMI 800, Snibe Diagnostics, Shenzhen, China). Anti-RBD IgG titers were expressed as arbitrary units per ml (AU/mL). Samples with values over 100 AU/mL were diluted and measured at 1:100, allowing extension of the dynamic range of analysis to 10,000 AU/mL. According to the manufacturer’s recommendations, a result greater or equal to 1 AU/mL was considered to be reactive.

In addition, we performed a fluorescence immunoassay (Ichroma™ COVID-19 nAb) for the measurement of SARS-CoV-2 neutralizing antibodies (Nab) to investigate the extent to which the anti-RBD antibodies, which may be present in the serum, are able to block the interaction between the RBD and ACE2 (angiotensin-converting enzyme). Results were interpreted as inhibition percentage (IH%), with a cut off positivity of IH ≥ 30%, according to the manufacturer.

#### 2.2.2. T-Cell Response

The T-cell response was evaluated via the QuantiFERON^®^ SARS-CoV-2 assay (Qiagen, Hilden, Germany), which is an interferon gamma release assay (IGRA). This assay uses three antigen tubes (SARS-CoV-2 Ag1, Ag2, and Ag3), containing a combination of specific SARS-CoV-2 peptide antigens that stimulate T cells. The QuantiFERON-SARS-CoV-2 Ag1 tube contains peptides derived from the S1 subunit (receptor binging domain) of the spike protein that stimulate CD4 T cells, and the Ag2 tube contains peptides from the S1 and S2 subunits of the spike protein that stimulate CD4 and CD8 T cell, while the Ag3 tube had additional peptides (covering the S, N, and M domains, as well as other peptides from proteins encoded by the full genome of SARS-CoV-2) to elicit a more complete specific T cell-mediated immune response. The test for the three antigens, at this time, were in vitro diagnostic products labeled for research use only (RUO) and were not yet validated for clinical purposes.

The amount of IFN-γ produced in response to SARSCoV-2-specific T cell stimulation was measured using an enzyme-linked immunosorbent assay (ELISA).

The kit also contains a positive control antigen (Mitogen) to confirm the proliferative capacity of patient lymphocytes at the time of sampling and a negative control “Nil tube” without stimulating peptides. Background IFN-ɣ levels of the negative control were subtracted from the raw values of stimulations to obtain the values used in the further analysis. Following the manufacturer’s recommendations, the QuantiFERON-SARS-CoV-2 was defined as positive if the IFN-γ level of SARS CoV-2 Ag, after background subtraction, was ≥0.15 IU/mL and ≥25% of Nil value.

### 2.3. Statistical Analysis

SPSS (IBM Statistics, version 20.0) and Prism (Graph Pad Software, version 8.0) were used for statistical analysis and data visualization. Categorical variables were expressed as frequency rates or percentages, and significance was detected by 𝜒2, or Fisher’s exact test. The Shapiro-Wilk normality test was conducted to estimate the distribution of the data, where *p* ˂ 0.05 indicate that RBD, Nab, and IFN-γ levels in our study were not normally distributed. Continuous variables were expressed as medians and interquartile [Q1–Q3] values. Differences between two independent groups were assessed using the Mann-Whitney U-test, and those between two dependent groups were assessed using the Wilcoxon test. The comparison of quantitative variables in multiples dependent groups was completed using the Friedman test, followed by the Wilcoxon signed-rank test for post-hoc analysis with Bonferroni adjustment, so as to compensate for multiple comparisons. Correlations were determined using the Spearman rank correlation analysis. Estimation of IGRA no-response risk was assessed by odds ratio (OR) and 95% confidence intervals (95% CI) by means of univariate logistic regression models. For all statistical analysis, *p* ˂ 0.05 was considered statistically significant.

## 3. Results

### 3.1. Humoral Response

**Figure 2:**
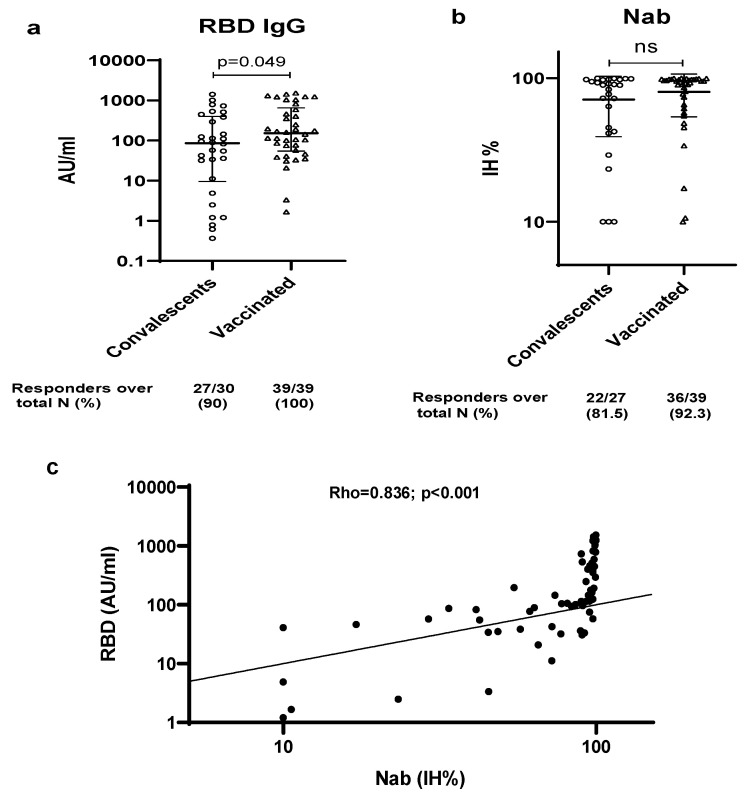
Evaluation of SARS-CoV-2 specific humoral response among convalescent and vaccinated subjects. (**a**) Anti-receptor binding domain (RBD) IgG (**b**) neutralizing antibody (Nab) (**c**) correlation between RBD and Nab. ns: not significant.

No IgG anti-RBDs were detected in the control group. All vaccinated subjects produced RBD-specific IgG at variable levels, whereas three individuals in the convalescent group had a negative result for IgG anti-RBD. Moreover, regarding the quantitative response, anti-RBD IgG concentrations were significantly higher among vaccinated subjects, as compared to convalescents (153 AU/mL [55–650.3] vs. 86 AU/mL [9.5–400.3], *p* = 0.049) (Figure 2a). 

The SARS-CoV-2 neutralizing antibodies were also measured for all individuals with anti-RBD IgG response (Figure 2b). Most cases of both groups observed a positive neutralizing antibodies capacity (81.5% of convalescent subjects and 92.3% of vaccinated ones). Regarding the percentage of inhibition, no significant difference was found between the two groups (89.1% [42.4–97.5] vs. 94% [65.2–97.7], *p* = 0.186). 

Overall, a strong positive significant correlation was found between neutralizing and anti-RBD IgG antibody levels (rho = 0.836, *p* ˂ 0.001) (Figure 2c).

### 3.2. SARS-CoV-2 Specific T-Cell Response

To determine a SARS-CoV-2 specific T cell reactivity, we measured IFN-γ response to SARS-CoV-2 peptides in the three groups. All participants showed robust IFN-ɣ release in response to the positive control (˃10 IU/mL). However, seven cases (four convalescent patients and three vaccinated cases) also had high IFN-ɣ levels in negative control tubes (˃8 IU/mL). These individuals were defined as having an indeterminate result and were excluded from further analysis. 

One of the healthy unvaccinated controls with negative serology for IgG anti-RBD had positive results in the IGRA (Ag3) test, implying a possible prior asymptomatic SARS-CoV-2 infection. 

**Figure 3:**
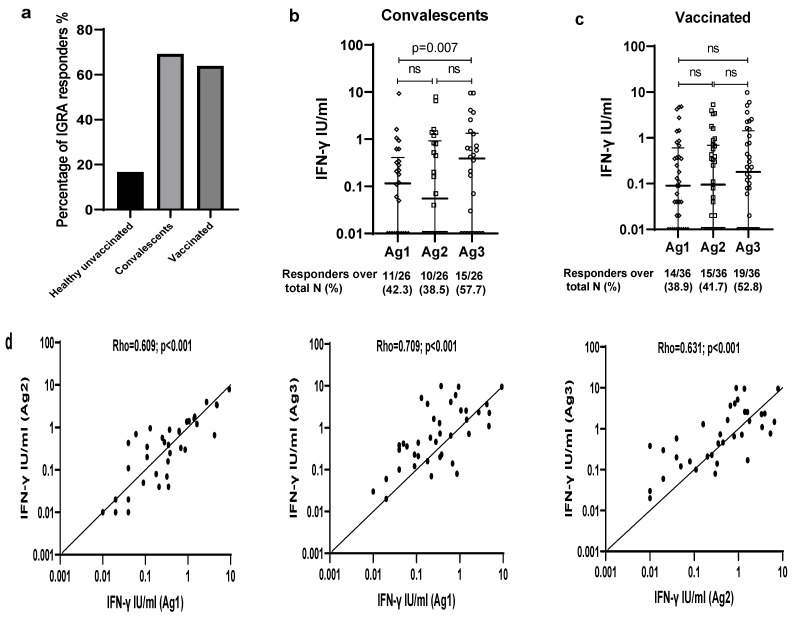

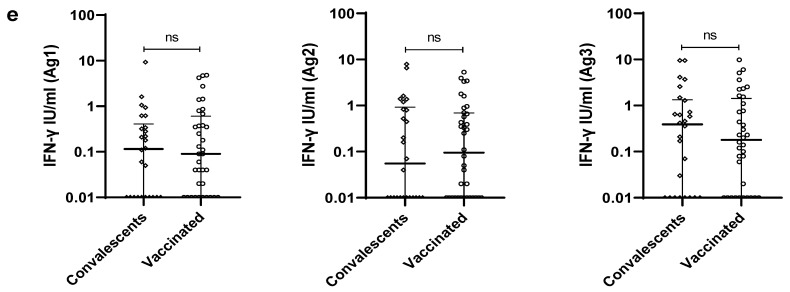
T cell response to QuantiFERON SARS-CoV-2. (**a**) Percentage of IGRA responders. (**b**) IFN-γ-specific T cell response to SARS-CoV-2 antigens among convalescents. (**c**) IFN-γ-specific T cell response to SARS-CoV-2 antigens among vaccinated subjects. (**d**) Correlations between the IFN-γ levels of all pairs of tested antigens. (**e**) Comparison of the levels of IFN-γ induced by each antigen between convalescent and vaccinated participants. ns: Not significant.

An elevated IFN-γ response in at least one Ag tube was observed in 69.2% (18/26) of convalescent subjects and 63.9% (23/36) of vaccinated subjects (Table 1, Figure 3a). Interestingly, in the three convalescent cases with negative IgG RBD, we detected a positive IGRA test after stimulation with Ag3, suggesting the development of a cellular immune response.

The rates of positivity to individual antigens were comparable between convalescent and vaccinated individuals (42.3% vs. 38.9% for Ag1, 38.5% vs. 41.7% for Ag2, 57.7% vs. 52.8% for Ag3, *p* > 0.05). Overall, among the three antigens, Ag3 demonstrated the highest rate of reactivity. Besides, in both groups, the majority of the IGRA responders reacted simultaneously to the 3 SARS-CoV-2 specific antigens (8/18 for convalescent subjects and 9/23 for vaccinated cases) (Table 1). 

Regarding the quantitative response, we found impressive individual differences in terms of IFN-ɣ released by the IGRA responders, varying from 0.18 to 9.32 IU/mL for Ag1, 0.16 to 7.82 IU/mL for Ag2, and 0.16 to 9.82 IU/mL for Ag3. When comparing the IFN-γ levels between the three antigen tubes, a significant difference was observed among convalescent subjects (*p* = 0.023). In particular, a higher IFN-γ response to Ag3 was detected, as compared to Ag1 (0.4 IU/mL [0–1.3] vs. 0.1 IU/mL [0–0.4]; *p* = 0.007) (Figure 3b). However, no significant difference has been found among vaccinated participants (*p* = 0.305) (Figure 3c). Overall, as shown in Figure 3d, positive significant correlations have been observed between the IFN-γ levels of all pairs of tested antigens. The strongest correlation was between IFN-γ response to Ag1 and Ag3 (rho = 0.709, *p* ˂ 0.001), followed by that between Ag2 and Ag3 (rho = 0.631, *p* ˂ 0.001) and Ag1 and Ag2 (rho = 0.609, *p* ˂ 0.001). Next, we compared the levels of IFN-γ induced by each antigen between convalescent and vaccinated participants. As illustrated in Figure 3e, no significant difference has been observed between the two groups.

To demonstrate any association between T-cell responses and vaccine type, vaccinated subjects were divided into two groups: those receiving inactivated vaccine (Sinovac; *N* = 26) and those receiving vector-based vaccines (Sputnik (*N* = 4) and Janssen (*N* = 6)). As shown in Table 2, no significant difference has been found between the two groups.

Finally, we focused on IGRA nonresponders—those who did not react to any of the SARS-CoV-2 antigens in both cohorts. The only factor that was associated with an absence of a cellular response at univariable analysis was time from blood collection, which was less than 30 days (OR:3.5, CI95% [1.15–10.50], *p* = 0.028) (Table 3). 

### 3.3. Evaluation of the Immune Response to SARS-CoV-2 Vaccine after Three-Month Follow Up

**Figure 4:**
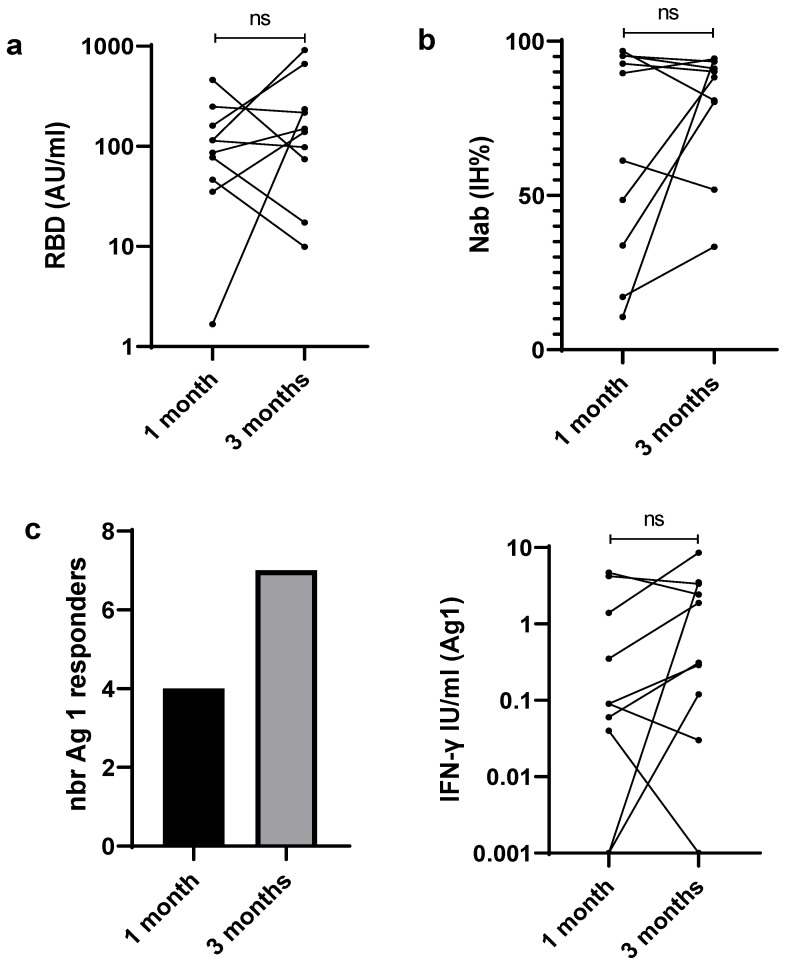

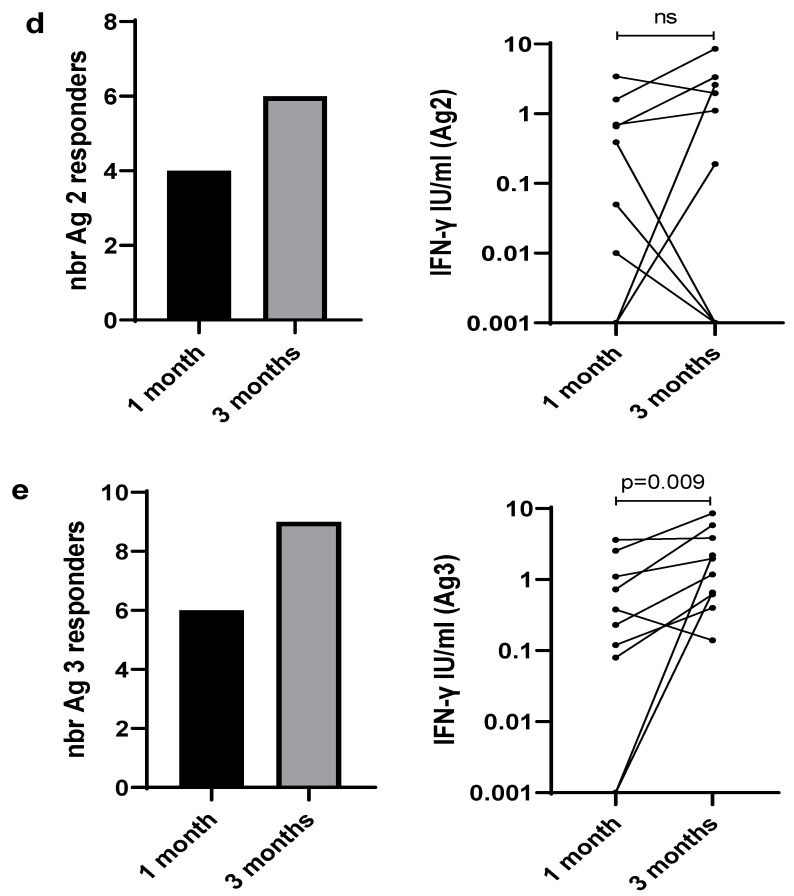
Follow up of humoral and cellular immune responses to SARS-CoV-2 after vaccination at two time points: one month and three months. (**a**) Anti-receptor binding domain (RBD) IgG. (**b**) Neutralizing antibody (Nab) (**c**) response to Ag1. (**d**) Response to Ag2. (**e**) Response to Ag3. nbr: number. ns: not significant.

Among the vaccinated subjects, 10 were evaluated both early (one month) and late (three months) after the second dose of the vaccine. RBD-IgG antibodies titers increased at three months in five patients and decreased in five others, and no significant difference has been found between the two points (Figure 4a). The same tendency was observed for Nab (Figure 4b). 

For the cellular response, as illustrated in Figure 4c–e, the rates of reactivity to SARS-CoV-2 antigens increased at three months, extending the number of IGRA responders from 7/10 at one month to 9/10 at three months. Moreover, a significantly higher IFN-γ response to Ag3 was detected at three months, as compared to initial evaluation (1.6 IU/mL [0.7–4.3] vs. 0.3 IU/mL [0.1–1.5]; *p* = 0.009). However, no significant difference was observed for Ag1 and Ag2 between the two time points. 

## 4. Discussion

Our results corroborate the published data supporting the QuantiFERON-SARS-CoV-2 as a useful diagnosis tool for monitoring the cellular immune response to the virus and for assessing the effectiveness of different vaccines. Indeed, the majority of participants, of both cohorts, had detectable IFN-γ-producing T-cell responses to at least one of the three tested SARS-CoV-2-specific peptide pools. Elevated levels of IFN-γ were observed in the three antigen tubes, suggesting that all are able to detect a T cell-mediated reactivity. Nevertheless, this reactivity was different between the starter and the extended set.

According to the manufacturer, the starter set include two antigen tubes: an Ag1 tube that contains CD4+ T cell epitopes from the S1 subunit of the spike protein and an Ag2 tube with both CD4+ and CD8+ T cell epitopes, derived from the S1 and S2 subunits of the spike protein. The rate of IGRA responders was quite similar after stimulation by Ag1 and Ag2. Moreover, quantitatively, no significant difference has been found between the level of IFN-γ induced by the two antigens. These finding suggest that IFN-γ specific T-cell response to spike was mainly mediated by CD4+ T cells in either post-COVID-19 subjects or vaccinated ones. To note, as seen in earlier reports, the pattern of cytokine production differed between the CD4+ and CD8+ subsets, according to protein specificity. In fact, prior studies in convalescents have shown that the M/NP (membrane/nucleocapsid proteins)-specific CD8+ had wider functionality than those factors targeting the spike protein [32], with higher IFN-γ production [33]. These data highlighted the importance of including non-spike proteins for a more accurate assessment of in vitro CD8+T cells’ functionality and thus elicit a more complete evaluation of the SARS-CoV-2 specific T-cell-mediated response. 

As expected, the Ag3 tube, which consisted of a selection of immunodominant peptides to the whole SARS-CoV-2 genome, demonstrated the highest rate of positivity in both cohorts, with a slightly higher quantitative response, as compared to Ag1 or Ag2 significance among convalescent individuals. Thus, the assay could be simplified to the use of just one single antigen, Ag3, notably, in post-COVID-19 subjects and those vaccinated with inactivated vaccine, was analyzed. In fact, natural infection and inactivated vaccines, which expose the immune system to the entire virus, elicit a broad immune response against different proteins on the virus. Interestingly, Jaganathan et al. reported a higher response to Ag3, even in individuals receiving spike-based vaccines [29].

Serological tests showed that, despite a PCR confirmed SARS-CoV-2 infection, the antibody test was negative for three convalescent subjects tested one month following COVID-19 diagnosis, while the IGRA test was positive. Similarly, a case within the control unvaccinated group, with no history of COVID-19 symptoms, had a detectable T-cell response in the absence of IgG-RBD antibodies, suggesting a possible previous SARS-CoV-2 asymptomatic infection. These results demonstrate that T cell reactivity may be detected in patients with asymptomatic or clinically mild disease in the absence of a seroconversion, as previously reported [8,9]. Nevertheless, we cannot rule out that some individuals of our cohorts may only have different specificities of circulating antibodies. This is supported by the result of the QuantiFERON-SARS-CoV-2 assay, showing a detectable Ag3 reactivity in the absence of Ag1 and Ag2 responses, indicating that, for these participants, T-cell response was not generated towards the spike protein. Thus, the Ag3 tube could show a particular interest in establishing the immune status of subjects seronegative for RBD-specific IgG. Correspondingly, Reynolds et al. [34] revealed that subjects lacking Nab tend to lack T-cell response to the spike protein while maintaining T-cell reactivity to other specificities. Taken together, the present data suggest the Ag3 tube as the most useful of the three antigens for evaluating SARS-CoV-2-specific T-cells’ response after infection and vaccination, as recently highlighted by Johnson et al. [31].

When comparing humoral to cellular response, we noticed that overall antibody seropositivity (nearly 96%) was higher than overall T-cell positivity (nearly 66%), leading to the question of how such disparity could be explained. The univariate analysis showed that timing in relation to infection or vaccination must be considered when interpreting QuantiFERON-SARS-CoV-2 results. Indeed, longitudinally, the QuantiFERON-SARS-CoV-2 assay detected an increase in the number of responders at three-month follow-up. This finding suggests that repeated testing could be appropriate in cases of a non-reactive QuantiFERON-SARS-CoV-2 result shortly after SARS-CoV-2 infection or vaccination. Kruse et al. led to the same conclusion for the T-SPOT.COVID test [35]. Nevertheless, Johnson et al. [31] reported a lower sensitivity of the essay when samples are gathered at a more distant time from infection (median 256, range 172–444 days) or vaccination (median 102, range 55–166 days). Therefore, more investigation is needed to establish the appropriate timeframe after infection or vaccination, during which the QuantiFERON-SARS-CoV-2 assay could be used to assess SARS-CoV-2 specific T cell response. 

Some limitations of the study need to be considered. Firstly, the inability to be certain that the vaccinated group participants were COVID-19-naives and had never been exposed to the SARS-CoV-2 virus given the endemic nature of the virus and the potential for asymptomatic infections is important. Additionally, for the control group and particularly for the case with a positive QuantiFERON-SARS-CoV-2 result, it is difficult to know whether it was an asymptomatic infection or a possible in vitro T cross-reactivity with other coronaviruses. Moreover, it is important to note that the vaccinated cohort mainly included subjects who received an inactivated vaccine, which elicit a broad response against different proteins of the virus. Thus, the findings cannot be generalized to other vaccine without further studies. Finally, the results of the study should be interpreted with caution because of the small sample sizes for different groups.

## 5. Conclusions

Overall, the inclusion of Ag3 improved the performance of the QuantiFERON-SARS-CoV-2. In fact, it detected T cell response in a higher proportion compared to the antigens derived from the spike domain. Moreover, the extended assay could constitute a valuable contribution to the evaluation of the immune response in subjects who fail to achieve a measurable antibody response after infection or vaccination. Nevertheless, further studies are needed to establish the appropriate timeframe after infection or vaccination during which the QuantiFERON-SARS-CoV-2 assay could be used to assess SARS-CoV-2-specific T-cell response and to validate the relevance of the present results for different types of vaccine.

## Figures and Tables

**Table 1:** Stratification of the data according to the response to SARS-CoV-2 antigens.

Responders	Convalescents (*N* = 26)	Vaccinated (*N* = 36)	*p*
At least one Ag; *n* (*n*/*N*%)	18 (69.2)	23 (63.9)	0.661
Ag 1; *n* (*n*/*N*%)	11 (42.3)	14 (38.9)	0.787
Ag 2; *n* (*n*/*N*%)	10 (38.5)	15 (41.7)	0.800
Ag 3; *n* (*n*/*N*%)	15 (57.7)	19 (52.8)	0.700
Only Ag1; *n* (*n*/*N*%)	2 (7.7)	1 (2.8)	/
Only Ag2; *n* (*n*/*N*%)	1 (3.8)	2 (5.6)	/
Only Ag3; *n* (*n*/*N*%)	5 (19.2)	4 (11.1)	/
Ag1 + Ag2; *n* (*n*/*N*%)	0 (0)	1 (2.8)	/
Ag1 + Ag3; *n* (*n*/*N*%)	1 (3.8)	3 (8.3)	/
Ag2 + Ag3; *n* (*n*/*N*%)	1 (3.8)	3 (8.3)	/
Ag1 + Ag2 + Ag3; *n* (*n*/*N*%)	8 (30.8)	9 (25)	/

**Table 2:** T-cell responses stratified by vaccine type.

Responders	Inactivated Vaccine (*N* = 26)	Vector-Based Vaccines (*N* = 10)	*p*
At least one Ag; *n* (*n*/*N*%)	16 (61.5)	7 (70)	0.716
Ag 1; *n* (*n*/*N*%)	12 (46.2)	2 (20)	0.255
Ag 2; *n* (*n*/*N*%)	11 (42.3)	4 (40)	1.000
Ag 3; *n* (*n*/*N*%)	13 (50)	6 (60)	0.717

**Table 3:** Univariate analysis of potential factors associated with IGRA no-response.

Factors	OR	95% CI	*p*
**Age**	1	[0.966–1.041]	0.880
**Gender (female)**	0.64	[0.213–1.914]	0.423
**Lymphocytes**	1	[0.999–1.001]	0.845
**Time from blood collection (** **˂** **30 days)**	3.47	[1.145–10.495]	**0.028**

## Data Availability

The datasets generated and analyzed during the current study are not publicly available due to health privacy concerns, but they are available from the corresponding author upon reasonable request.
